# Relation between Body Structure and Hearing during Soft Tissue Auditory Stimulation

**DOI:** 10.1155/2015/172026

**Published:** 2015-04-16

**Authors:** Cahtia Adelman, Michal Kaufmann Yehezkely, Shai Chordekar, Haim Sohmer

**Affiliations:** ^1^Speech & Hearing Center, Hebrew University-Hadassah Medical Center, P.O. Box 12000, 91120 Jerusalem, Israel; ^2^Department of Communication Disorders, Hadassah Academic College, 37 Hanevi'im Street, P.O. Box 1114, 91010 Jerusalem, Israel; ^3^Department of Otorhinolaryngology/Head & Neck Surgery, Hebrew University-Hadassah Medical Center, P.O. Box 12000, 91120 Jerusalem, Israel; ^4^Department of Communication Disorders, Sackler Faculty of Medicine, Tel Aviv University, Ramat Aviv, P.O. Box 39040, Tel Aviv, Israel; ^5^Department of Medical Neurobiology (Physiology), Institute for Medical Research Israel-Canada, Hebrew University-Hadassah Medical School, P.O. Box 12272, 91120 Jerusalem, Israel

## Abstract

Hearing is elicited by applying the clinical bone vibrator to soft tissue sites on the head, neck, and thorax. Two mapping experiments were conducted in normal hearing subjects differing in body build: determination of the *lowest* soft tissue stimulation *site* at which a 60 dB SL tone at 2.0 kHz was effective in eliciting auditory sensation and assessment of actual *thresholds* along the midline of the head, neck, and back. In males, a lower site for hearing on the back was strongly correlated with a leaner body build. A correlation was not found in females. In both groups, thresholds on the head were lower, and they were higher on the back, with a transition along the neck. This relation between the soft tissue stimulation site and hearing sensation is likely due to the different distribution of soft tissues in various parts of the body.

## 1. Introduction

In addition to hearing by air conduction (AC) and by bone conduction (BC), hearing sensation can also be elicited when stimulation is applied to soft tissue sites on the head, neck, and thorax [1–7]. This mode of stimulation has also been called soft tissue conduction (STC) or nonosseous BC, due to the fact that the standard clinical bone vibrator can elicit hearing even when the vibrator is applied to sites not overlying skull bone [8, 9]. Others have referred to the same mode as body conduction, based on the finding that the noise field during magnetic resonance imaging can still be heard even in the presence of earplugs, earmuffs, and a helmet used together, suggesting that the noise heard by the subject under those conditions is conducted mainly through the body [2].

In the present report, this additional mode will be called nonosseous BC or STC. In a previous study on STC, subjects were able to hear when pure tones were delivered by soft tissue stimulation to points along the midline of the back over the vertebra, and it seemed that subjects with a leaner body structure reported auditory sensation to stimulation at sites lower down on back than subjects with an obese build [10]. The present more detailed mapping study was designed to assess whether there is a correlation between some aspects of body structure and the lowest (farthest from the ears) point on the midline of the body at which normal hearing subjects report auditory sensation in response to STC stimulation. Men and women differ in percentage and distribution of adipose tissue and muscle [11]. Therefore, two separate groups of male and female subjects were tested. In these subjects, we determined the lowest point at which auditory sensation could be elicited at well-defined anatomical sites: the vertical midline of the back, at which the bone vibrator is delivering the same uniform sound intensity, elicited auditory sensation in males and females. In addition, several measures of body structure were assessed, together with the correlation between these measures and the lowest point on the back at which auditory sensation was elicited.

The possible relation between hearing and body structure was also assessed within subjects: actual auditory thresholds were determined when the bone vibrator was applied to a series of anatomical sites at the midline. These included sites on the skin along segments of the body at which there are transitions in body structure: over the larger volume of the thorax to sites over the smaller volume of the head and over the transition region from the head to the thorax along the cervical vertebra of the neck.

The rationale for this study on soft tissue conduction was to test the hypothesis that a correlation exists between the overall distribution of soft tissues in the body and the sites at which hearing can be elicited. Furthermore, such an analysis can provide further insight into the mechanisms of STC, enhancing its relevance.

## 2. Methods

### 2.1. General

Subjects were adults with normal hearing, defined as air conduction thresholds to 500, 1000, 2000, and 4000 Hz at 15 dB HL or better bilaterally (ANSI). The experiments were conducted with an AC 40 clinical audiometer (Interacoustics, Denmark). The B71 standard clinical bone vibrator was applied by the same experimenter by hand to all skin sites with a uniform static pressure of 500 gram (5 Newton). The 5 N force was achieved by manually pressing down onto a 1.2 cm diameter metal spring to the same extent that a weight of 500 gram (5 N force) compressed the spring. The spring was attached at its other end to the surface of the bone vibrator opposite to that in contact with the skin. This 5 N force is similar to the standard application force (ANSI S3.6–5.4 N ± 0.5 N) provided by the Radioear P3333 headband of the clinical bone vibrator and is more convenient than the head band for application to sites not on the head. Use of such a spring to uniformly deliver the 5 N force has been reported previously [10].

The stimulus was a 2000 Hz warble tone since at this frequency there is no occlusion effect [12, 13] and there is no vibratory tactile sensation [14]. Throughout the testing, the subjects were equipped with foam earplugs (classic SuperFit 30 Aero Co. E-A-R) deeply inserted bilaterally and with earphones serving as ear protectors in order to further block air conducted sound to ensure that they would respond to the STC stimuli and not to AC sounds coming from the bone vibrator. As a further control for this possibility, thresholds were also obtained with the bone vibrator held in the air over the various sites but not touching the skin to be sure that these were always higher (at times as much as 50 dB higher) than that with the bone vibrator pressed to skin at that site.

### 2.2. Experiment  1

The experiment is about farthest site of stimulation for hearing on the back. Ten males (mean ± SD age 32 ± 9 years, range 25–55 years) and fifteen females (mean ± SD age 26 ± 8 years, range 18–50 years) participated in this part of the study. An experimenter (otorhinolaryngologist/head and neck surgeon) applied the bone vibrator manually with a constant pressure of about 500 gram to the skin over various vertebrae determined by surface palpation of the spinous process. This method of surface palpation has been shown to be accurate within one spinous process (vertebra) [15]. In this experiment, the lowest (farthest from the ear) skin site on the midline of the back (over the vertebrae) at which the 2000 Hz tone at a constant intensity of 60 dB SL was audible to each subject was determined. The fairly loud stimulus intensity of 60 dB SL (sensation level, i.e., level above threshold of the subject) was used so as to have as wide a range of body sites as possible, so that the individual hearing threshold of each subject would be less likely to affect the results. We also determined the lowest point one centimeter lateral (on the same side as the mastoid studied) to the midline spinal column at which an auditory sensation was reported under the same conditions. In order to control for possible air conducted sounds coming from the bone vibrator, thresholds were determined when it was held in the air above the lowest points. Furthermore, for each subject, weight, height, neck circumference (just below the larynx), waist circumference (point of minimal abdominal circumference, usually located about halfway between the navel and the lower end of the sternum in females; in males, across the navel), and in women hip circumference (over the greatest protrusion of the gluteus muscles) were measured. Using these measurements, body mass index (BMI, defined as weight in kg divided by height in meters^2^) and percent body fat (US Navy procedure-Department of Defense Instruction number 1308.3, Nov. 5, 2002) were determined based on calculations involving weight, height, neck circumference, waist circumference, and in women hip circumference.

### 2.3. Experiment  2

The experiment is about actual threshold determinations at body segment transitions. The subjects in this separate group were nine females aged 20–38 years (mean ± SD, 25.2 ± 5.5 years) and eight males aged 26–37 years (29.9 ± 3.4 years). Behavioral hearing thresholds to a warble tone of 2000 Hz were determined with the bone vibrator applied with an application force of 5 N to the skin over both mastoids, and thresholds at the additional sites were expressed in dB relative to that at the mastoid, since the threshold was lowest at the mastoid, and the mastoid is a standard BC testing site. The midline skin sites at which thresholds to the bone vibrator applied with the same 5 N spring were determined. They were mastoid, vertex, occiput, inion (the occipital protuberance), cervical vertebra 1, 2, 3, 4, 5, 6, and 7, and thoracic vertebra 2, 4, 8, and 12. Since differences were found between males and females in experiment  1, results of the two groups (males and females) were evaluated separately. The experimental protocol was reviewed and approved by the Hadassah Academic College Institutional Ethics Committee, and subjects gave their informed consent.

## 3. Results

### 3.1. Experiment  1 (Males): Lowest Point of Hearing on the Back

**Figure 1:**
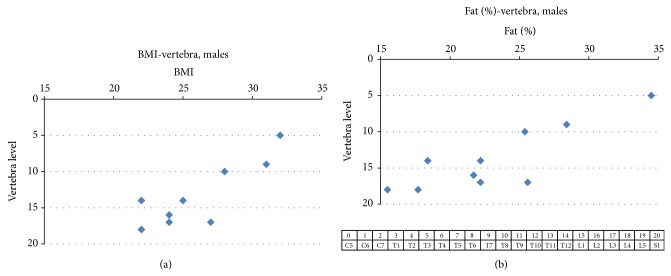
(a) shows the relation between BMI and the lowest vertebra (vertebrate level defined at bottom of figure) at which auditory sensation was elicited in response to 2.0 kHz tones at 60 dB SL in 10 normal male subjects. It can be seen that the lowest vertebra eliciting hearing sensation ranged from T3 to L4. The point on the graph at BMI 22/T10 represents two points with identical values. The Spearman rank order correlation coefficient for this relation was rs = 0.78. (b) shows the relation between percent of body fat and the lowest vertebra (rs = 0.76) in males. In all of these experiments, the subjects were equipped with ear plugs to reduce hearing by AC.

**Figure 2:**
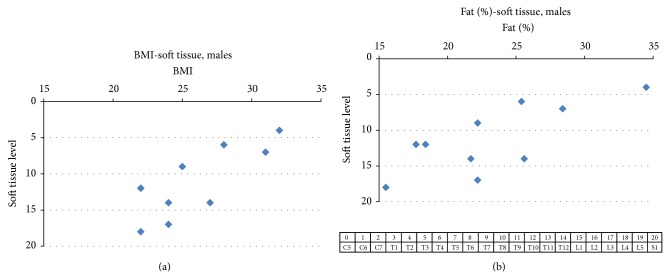
(a) shows the relation between BMI and lowest point on the soft tissue one centimeter to the side of the spine (expressed as vertebra level defined at bottom of figure) at which auditory sensation was elicited in response to 2.0 kHz tones at 60 dB SL in 10 normal male subjects. The point on the graph at BMI 22/L4 represents two points with identical values. The Spearman rank order correlation coefficient for this relation was 0.70. (b) shows the relation between percent of body fat and lowest point (rs = 0.64) in males.

It can be seen in Figure 1 that the midline anatomical skin site over the lowest vertebra at which auditory sensation at 60 dB SL was elicited in different subjects ranged from T3 to L4. The BMI of the ten men ranged from 22 to 32 kg/m² and percent body fat ranged from 15.5 to 34.5. In the male subjects with lower BMI and percent body fat (i.e., leaner subjects), the lowest stimulation site at which sensation was reported was further down on the back. Significant nonparametric correlations (Spearman rank order) were found for the relation between the lowest vertebra at which auditory sensation was elicited and BMI (rs[10] = 0.78, *P* < 0.01), between percent body fat (rs[10] = 0.76, *P* < 0.01), weight (rs[10] = 0.77, *P* < 0.01), and waist circumference (rs[10] = 0.76, *P* < 0.01) but not for height (rs[10] = 0.04, *P* = 0.89) and not for neck circumference (rs[10] = 0.56, *P* = 0.08). The lowest region on the soft tissue one centimeter lateral to the midline at which auditory sensation was elicited, expressed in terms of the corresponding parallel vertebra level, ranged from T2 to L4 (shown in Figure 2) and was either rostral to the lowest vertebra at which sound was heard or at the same level depending on the subject but never lower than the lowest vertebra at which sensation of hearing was reported. Similar to the findings at midline points, significant correlations were found between the lowest soft tissue point at which auditory sensation could be elicited and BMI (rs[10] = 0.70, *P* < 0.05) and for body fat percentage (rs[10] = 0.64, *P* < 0.05) (shown in Figure 2), weight (rs[10] = 0.85, *P* < 0.0001), neck circumference (rs[10] = 0.74, *P* < 0.05), and waist circumference (rs[10] = 0.80, *P* < 0.01) but not for height (rs[10] = 0.2, *P* = 0.4).

### 3.2. Experiment  1 (Females): Lowest Point of Hearing on the Back

The lowest vertebra at which auditory sensation was elicited at 60 dB HL ranged from T9 to S1. The BMI of the fifteen females ranged from 19 to 32 kg/m² and percent body fat ranged from 23.6 to 45.9. The correlation (Spearman rank order) between the lowest vertebra at which auditory sensation was elicited and BMI was not significant (rs[15] = 0.24, *P* = 0.37), and it also wasn't significant for body fat percentage (rs[15] = −0.04, *P* = 0.88). Also, the correlations between the lowest soft tissue point lateral to the vertebrae at which auditory sensation was elicited and BMI were not significant (rs[15] = 0.23, *P* = 0.39) as was the case for body fat percentage (rs[15] = 0.14, *P* = 0.6). This absence of correlation in females led us to compare correlations also in two subgroups of the original study sample (*N* = 6 each), 6 males and 6 females matched for identical BMIs, over a wide range of BMIs. In these subgroups also, a correlation was again found in males but not in females. Since there was no correlation, the graphs are not presented.

### 3.3. Experiment  2: Actual Threshold Determinations at Body Segment Transitions

**Figure 3:**
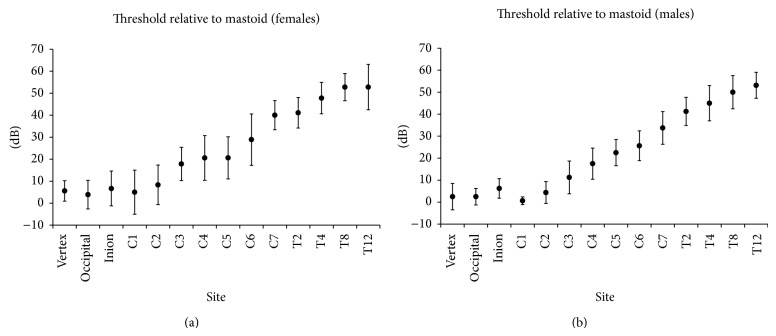
Mean (±1SD) threshold in dB relative to that at the mastoid along midline sites on the head, neck, and back in 9 females (a) and in 8 males (b) with normal hearing.

The mean (±SD) thresholds relative to that at the mastoid at the different anatomical midline sites are displayed in Figure 3 for females (Figure 3(a)) and for males (Figure 3(b)). Two-way repeated measures ANOVA separated the main effects of gender and site. ANOVA indicated significant effect of gender [*F*(1,230) = 7.437, *P* = 0.0007] and site [*F*(13,230) = 108.174, *P* < 0.001] but didn't indicate significant interaction between gender and site [*F*(13,230) = 0.292, *P* = 0.993]. In both groups, overall, the thresholds were higher when measured further from the head: mean thresholds over the thoracic vertebra were generally 40–50 dB higher than those at the mastoid in both groups; on the head sites, thresholds were only 5–7 dB greater than at the mastoid. Over the cervical vertebra, there was a transition from 5 dB at C1 to 40 dB at C7 in females and 1 to 34 dB in males.

## 4. Discussion

Following the demonstration that auditory sensation can be elicited by applying the bone vibrator to the head, neck, and thorax (STC) [10], the present study is an attempt to assess the possibility that some aspects of these sensations are correlated with distribution of body structure and size. The results show that there is a significant correlation in males between the midline site over the spinal column most distant from the ear at which a male subject still heard the 2.0 kHz tone at 60 dB SL and several measures of the body structure of the subject. Subjects with leaner body build, for example, lower BMI and percent body fat, were able to hear the tone lower down on the back. Note that this was independent of the height of the male subjects.

Interestingly, such a correlation was not found in the female subjects. The reason for the presence of correlation between body build and the soft tissue stimulation site most distant from the ear in males and the absence of it in females is not clear. However, although gender differences with respect to bone mass have been shown for example [16], it is likely that this diversion between males and females is more related to the differing overall distribution of soft tissues, for example, adipose and muscle, in the body between males and females [11].

These correlations in males (and not in females) were apparent when the intensity of the 2.0 kHz sound stimulus was 60 dB SL. It is possible that, had we used different intensities, the correlation could have been different.

Thus, it is possible that the STC auditory sensation elicited at the STC sites is related in one way or another to some aspect of body structure, since, as is apparent from the results of experiment 2 (thresholds along the midline of the head, neck, and back), the thresholds are uniformly lowest on the head and uniformly highest along the back in males and in females. However, this relation is not a simple linear function of the distance between the stimulation site and the ear. There is a clear transition along the neck from the generally lower thresholds on the head to the overall higher thresholds on the back. Furthermore, no correlation was found between the lowest stimulation site on the back (farthest from the ear) at which the subjects heard the 60 dB SL tone and the height of the subjects. It is likely that these findings are a result of the nature of the pathway from the soft tissue stimulation site along the tissues of the head, neck, and thorax to the inner ear eliciting the auditory sensation. Also, it is not likely that the vibrations induced in the tissues of the thorax could cause vibrations of the relatively distant skull bone, which would be required to lead to the major classical mechanisms of bone conduction: ossicular chain inertia, cochlear compression-distortion, and cochlear fluid inertia [17, 18]. This is due to the distance and to the difference in acoustic impedance between the tissues. Furthermore, the auditory nerve brainstem evoked response thresholds to STC stimulation in experimental animals are not altered by manipulations which interfere with the classical mechanisms of skull bone conduction, such as fixation of the ossicular chain and the two windows and discontinuity of the ossicular chain [19]. In addition, threshold intensity STC stimulation did not induce laser Doppler vibrometry detected vibrations of skull bone [20]. Thus the different STC thresholds over the neck and thorax are probably not due to mechanisms involving vibrations of skull bone conduction. It is more likely that the tissue vibrations induced by the bone vibrator at the soft tissue sites are transmitted along tissues with similar acoustic impedances from the initial soft tissue stimulation site to the inner ear [21]. Thus the presence of different proportions of soft tissues and their distribution and the physical dimension of body parts can have an effect on the magnitude (intensity) of the acoustic frequency vibrations which finally reach and activate the inner ear. It is likely that the vibrations induced lower down on the back would be dispersed in the volume of the body between the site of their induction and the inner ear. Therefore, subjects with a thin body build (low BMI, low fat %) would be able to hear when stimulated at soft tissue sites lower down on the back compared to an obese subject. Furthermore, when actual thresholds were determined along midline sites on the head, back of the neck, and thorax; lower thresholds were observed when the STC stimulation was applied to the smaller volume of the head (less dispersion) both in females and males, higher thresholds were observed when the STC stimulation was applied to the larger thorax (greater dispersion of the vibratory energy), and a gradual transition of thresholds was observed when the STC stimulation was applied to the neck (a “transition zone” between the smaller head and the larger thorax) in all subjects.

Overall, these results provide evidence for a relation between the auditory sensations elicited in response to nonosseous BC (STC) stimulation and the anatomical site of such stimulation as a result of the distribution of tissues in different parts of the body. It has been suggested that nonosseous BC (STC) stimulation can contribute to the differential diagnosis between a conductive hearing loss and a sensorineural loss since it does not involve middle ear structures and reflects true cochlear function [21]. Therefore, these findings have implications for the possible clinical use of nonosseous BC (STC) in such diagnosis and in choice of the stimulation site in cases where BC stimulation is not appropriate, as in severe skull fractures, wide spread head hematomas, mastoiditis, or abscess at these sites, especially in children.

## References

[1] Sohmer H., Freeman S., Geal-Dor M., Adelman C., Savion I. (2000). Bone conduction experiments in humans—a fluid pathway from bone to ear. *Hearing Research*.

[2] Ravicz M. E., Melcher J. R. (2001). Isolating the auditory system from acoustic noise during functional magnetic resonance imaging: examination of noise conduction through the ear canal, head, and body. *Journal of the Acoustical Society of America*.

[3] Seaman R. L. (2002). Non-osseous sound transmission to the inner ear. *Hearing Research*.

[4] Berger E. H., Kieper R. W., Gauger D. (2003). Hearing protection: surpassing the limits to attenuation imposed by the bone-conduction pathways. *Journal of the Acoustical Society of America*.

[5] Watanabe T., Bertoli S., Probst R. (2008). Transmission pathways of vibratory stimulation as measured by subjective thresholds and distortion-product otoacoustic emissions. *Ear and Hearing*.

[6] Ito T., Röösli C., Kim C. J., Sim J. H., Huber A. M., Probst R. (2010). Bone conduction thresholds and skull vibration measured on the teeth during stimulation at different sites on the human head. *Audiology and Neurotology*.

[7] Sinha T., Lenhardt M. L. (2011). Eyes as windows on brain pressure. *International Tinnitus Journal*.

[8] Vento B. A., Durrant J. D., Katz J., Burkard R., Hood L., Medwetsky L. (2009). Assessing bone conduction thresholds in clinical practice. *Handbook of Clinical Audiology*.

[9] Chordekar S., Kriksunov L., Kishon-Rabin L., Adelman C., Sohmer H. (2012). Mutual cancellation between tones presented by air conduction, by bone conduction and by non-osseous (soft tissue) bone conduction. *Hearing Research*.

[10] Kaufmann M., Adelman C., Sohmer H. (2012). Mapping sites on bone and soft tissue of the head, neck and thorax at which a bone vibrator elicits auditory sensation. *Audiology and Neurotology Extra*.

[11] Jackson A. S., Stanforth P. R., Gagnon J. (2002). The effect of sex, age and race on estimating percentage body fat from body mass index: the Heritage Family Study. *International Journal of Obesity and Related Metabolic Disorders*.

[12] Tsai V., Ostroff J., Korman M., Chen J. M. (2005). Bone-conduction hearing and the occlusion effect in otosclerosis and normal controls. *Otology and Neurotology*.

[13] Dean M. S., Martin F. N. (2000). Insert earphone depth and the occlusion effect. *The American Journal of Audiology*.

[14] Hyvarinen J., Sakata H., Talbot W. H., Mountcastle V. B. (1968). Neuronal coding by cortical cells of the frequency of oscillating peripheral stimuli. *Science*.

[15] Harlick J. C., Milosavljevic S., Milburn P. D. (2007). Palpation identification of spinous processes in the lumbar spine. *Manual Therapy*.

[16] Nieves J. W., Formica C., Ruffing J. (2005). Males have larger skeletal size and bone mass than females, despite comparable body size. *Journal of Bone and Mineral Research*.

[17] Stenfelt S., Goode R. L. (2005). Bone-conducted sound: physiological and clinical aspects. *Otology and Neurotology*.

[18] Stenfelt S. (2011). Acoustic and physiologic aspects of bone conduction hearing. *Advances in Oto-Rhino-Laryngology*.

[19] Perez R., Adelman C., Chordekar S., Ishai R., Sohmer H. (2014). Air, bone and soft tissue excitation of the cochlea in the presence of severe impediments to ossicle and window mobility. *European Archives of Oto-Rhino-Laryngology*.

[20] Chordekar S., Adelman C., Sohmer H. (2014). Bone conduction thresholds without bone vibrations. *Journal of Basic and Clinical Physiology and Pharmacology*.

[21] Adelman C., Cohen A., Regev-Cohen A., Chordekar S., Fraenkel R., Sohmer H. (2015). Air conduction, bone conduction, and soft tissue conduction audiograms in normal hearing and simulated hearing losses. *Journal of the American Academy of Audiology*.

